# Supplementary material for: Enhanced Deformability Through Distributed Buckling in Stiff Quasicrystalline Architected Materials
Source: Adv Mater. 2025 Apr 14;37(24):2505125. doi: 10.1002/adma.202505125 (PMC12177859; doi:10.1002/adma.202505125)
Supplement: Supplementary file 1 — Supporting Information [file ADMA-37-2505125-s001.pdf]

ADVANCED MATERIALS

Supporting Information

for *Adv. Mater.*, DOI 10.1002/adma.202505125

Enhanced Deformability Through Distributed Buckling in Stiff Quasicrystalline Architected Materials

Matheus I. N. Rosa, Konstantinos Karapiperis, Kaoutar Radi, Elias Pescialli and Dennis M. Kochmann**

Enhanced Deformability Through Distributed Buckling in Stiff Quasicrystalline Architected Materials: Supporting Information

Matheus I. N. Rosa, Konstantinos Karapiperis, Kaoutar Radi, Elias Pescialli, Dennis M. Kochmann**

Dr. M. I. N. Rosa, Dr. K. Radi, E. Pescialli, Prof. D. M. Kochmann
Department of Mechanical and Process Engineering
ETH Zürich, Zurich, Switzerland
Email Address: minguaggiato@ethz.ch, dmk@ethz.ch
Prof. K. Karapiperis
School of Architecture, Civil and Environmental Engineering
EPFL, Lausanne, Switzerland

Supporting note 1: Generation of 3D Quasicrystalline Trusses

We create 3D icosahedral quasicrystal lattices by adopting the cut-and-project method applied to a 6D simple cubic lattice [1, 2]. The cubic lattice points $\mathbf{X}_h$ in the high dimensional (6D) space are defined by vectors of integer coordinates $\mathbf{X}_h = [i_1, i_2, ..., i_6]$, assuming a unitary lattice constant for simplicity. The projection is realized via a projection matrix, which can be expressed as [2]:

$$\mathbf{M} = \frac{1}{\sqrt{1+\tau^2}} \begin{pmatrix} \tau & \tau & 0 & -1 & 0 & 1 \\ 0 & 0 & 1 & \tau & 1 & \tau \\ 1 & -1 & -\tau & 0 & \tau & 0 \\ \tau & -\tau & 1 & 0 & -1 & 0 \\ -1 & -1 & 0 & -\tau & 0 & \tau \\ 0 & 0 & \tau & -1 & \tau & -1 \end{pmatrix}, \tag{1}$$

where $\tau = (1+\sqrt{5})/2$ is the golden ratio. The projected lattice coordinates $\mathbf{X}_p$ are then obtained through the relation $\mathbf{X}_p = \mathbf{M}\mathbf{X}_h$. The first three components of $\mathbf{X}_p$ represent positions in the 3D physical space, while the remaining three components define positions in the perpendicular 3D complementary (or internal) space. Not all lattice points originating from the hyper-space lattice are accepted as lattice points in the 3D physical space. Instead, a selection is performed through an acceptance domain (or window) in the internal space, which is defined by the projection of a Wigner-Seitz cell of the 6D simple cubic lattice onto the internal space.

The projection procedure is illustrated in Figure S1 for a hypercubic lattice of size 3 centered at the origin, i.e. with $\mathbf{X}_h$ having vector components in the set $[-1, 0, 1]$. Figure S1a illustrates the selection window in internal space for a Wigner-Seitz cell centered at the origin of the 6D hyper-space lattice, whose convex hull defines a rhombic triacontahedron [3]. The quasicrystal lattice in 3D physical space is defined by points whose internal space components lie within the window (blue dots in the figure), while points outside (gray) are excluded. The lattice points are connected by a strut if their high-dimensional space counterparts are also connected in the 6D cubic lattice. However, we make one important distinction with respect to previous works [1, 2] and do not include points whose projections are on the boundary of the acceptance window (red points in Figure S1a). If accepted, these points lead to intersecting struts, as illustrated in Figure S1b. Our choice instead leads to a lattice without any intersecting members, as illustrated in Figure S1c. The exclusion of points which lie on the surface of the acceptance domain is achieved by applying an infinitesimal contraction to the Wigner-Seitz cell of the 6D simple cubic lattice.

The creation of the dual trusses requires the definition of the polyhedron components of the icosahedral quasicrystal lattice, which are illustrated in Figure S2. The cut-and-project method typically generates a 3D tiling of only two constituents: the oblate and the prolate rhombohedra, displayed in Figs. S2(a,b).

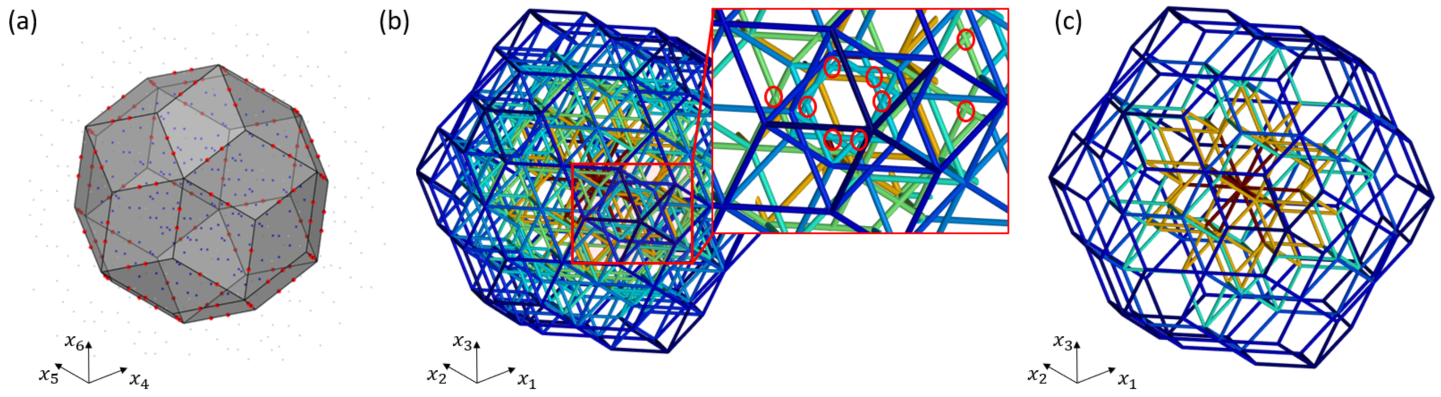

Fig. S1: Cut-and-project generation of quasicrystal lattices. (a) The selection window in internal space. (b,c) Icosahedral quasicrystal lattice by including or not including the points on the boundary of the acceptance window (red in (a)). Inclusion of such points lead to intersecting struts, as highlighted in (b). Beams are colored by their distance to the center of the lattice to aid visualization.

The inclusion of points on the boundaries of the acceptance window produces such a tiling. However, as previously mentioned, struts may intersect (Figure S1), which was also reported in [4] (cf. their Figure 5b). By excluding the boundary of the window, the lattice is composed of two additional polyhedra: the rhombic dodecahedron (Figure S2(c)) and the rhombic icosahedron (Figure S2(d)). We illustrate the polyhedra composition of the icosahedral quasicrystal considered here for increasing domain sizes in Figure S2(e). The presence of more than two polyhedra is also commonly found in icosahedral quasicrystals created through substitution rules [5] or through the multi-grid method [6, 7]. The regular quasicrystal truss is defined by taking the edges of the polyhedra, while the dual truss is defined by connecting the centroids of adjacent polyhedra, as illustrated in the main text.

Supporting note 2: Elastic simulations

This section presents additional numerical results to support the description provided in the main text. Figure S3 displays the distribution of strain energy density for the simulated 3D trusses, corresponding to the linear regime results of Figure 3. The histograms show concentrated distributions of axial strain energy in stretching-dominated periodic designs (cubic, octet, and isotropic lattices), which correlates with uniform force chains that lead to global buckling. In contrast, the icosahedral dual lattice is characterized by a non-uniform distribution of force chains, reflected in the dispersed stretching strain energy histogram and correlating with the stable nonlinear deformation characterized by localized instabilities. Both the Kelvin and icosahedral lattices are dominated by bending strain energy, in agreement with their bending-dominated character.

To illustrate the isotropic behavior of the quasicrystalline designs (and the anisotropy of others), Figure S4 compares nonlinear responses of 2D and 3D trusses when compressed along different directions. Simulations were conducted by maintaining the vertical compression and reorienting the lattices by the indicated directions, followed by a truncation within the same square (2D) and cubic (3D) domains. Solid lines correspond to the compression direction presented in the main text. One additional direction is chosen according to the symmetry of each particular lattice to illustrate their direction-dependent response (crossed lines). In 2D, the square lattice presents the most anisotropic response, with the compression along the diagonal direction ($\theta = \pi/4$) showing a compliant bending-dominated behavior. The hexagonal and triangular lattices are both linearly isotropic and therefore maintain the same stiffness (linear slope) when compressed along the $\theta = \pi/6$ direction. However, their isotropy is not maintained in the nonlinear regime, where the triangular lattice shows two different buckling plateaus, while the hexagonal lattice shows deviations in the nonlinear response that steadily increase with strain. The two quasicrystal lattices show a large degree of isotropy. The Penrose lattice shows almost indistinguishable nonlinear responses for the two directions considered, while the Penrose dual lattice shows small deviations primarily in the

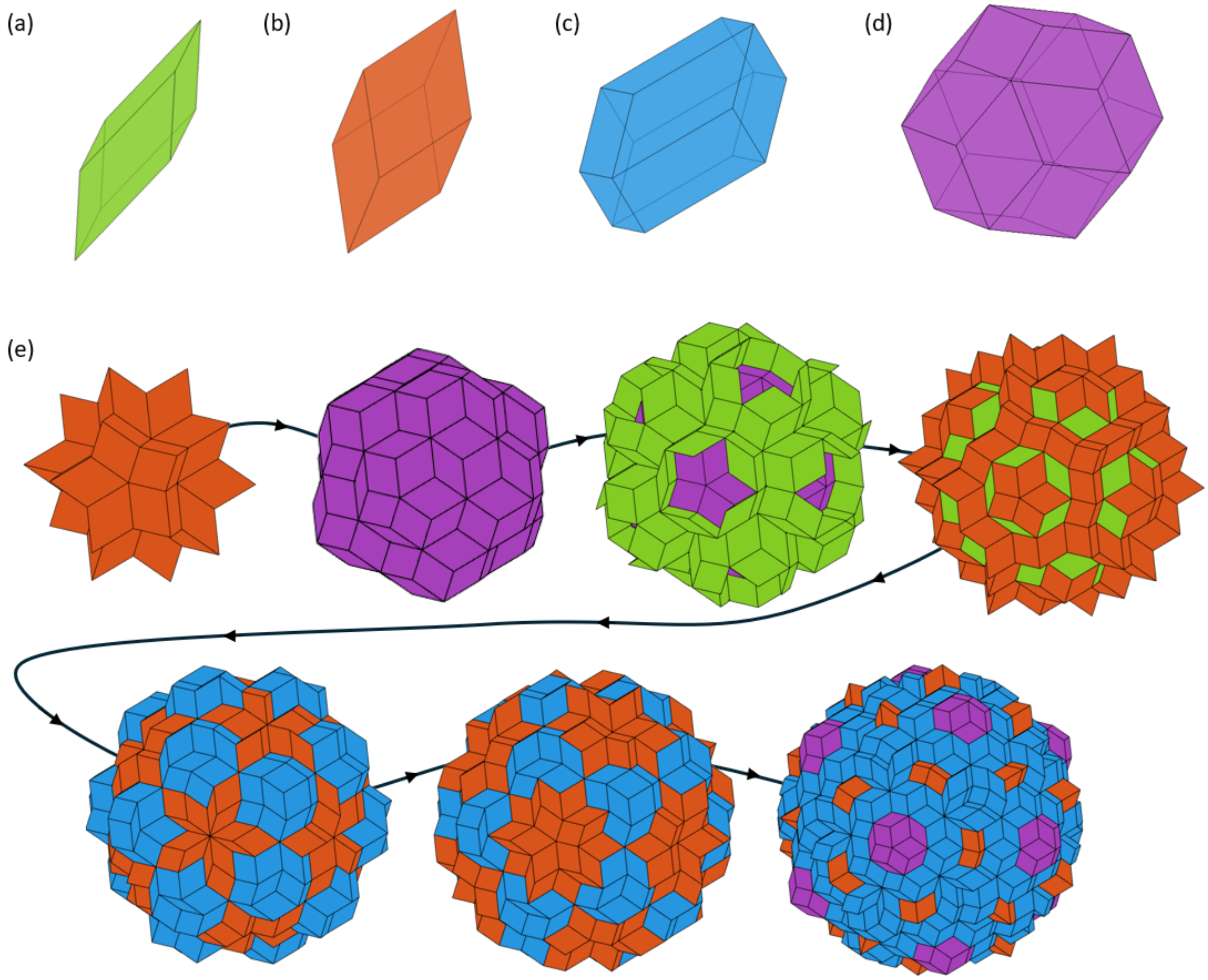

Fig. S2: Icosahedral quasicrystals and their polyhedra composition of (a) the oblate rhombohedron, (b) the prolate rhombohedron, (c) the rhombic dodecahedron, and (d) the rhombic icosahedron. (e) Visualization of the 3D tile for increasing domain sizes.

buckling plateau but very similar (and hence approximately direction-independent) nonlinear responses.

In 3D, the results presented in the main text consider compression along the z axis, which corresponds to the standard [001] orientation for periodic lattices, and to an axis of $\mathcal{C}_2$-symmetry for the quasicrystals (as shown in Figure 1). The additional results presented in Figure S4(b) (crossed lines) consider compression along the diagonal orientation [111] for periodic lattices, and along one axis of $\mathcal{C}_5$-symmetry for the

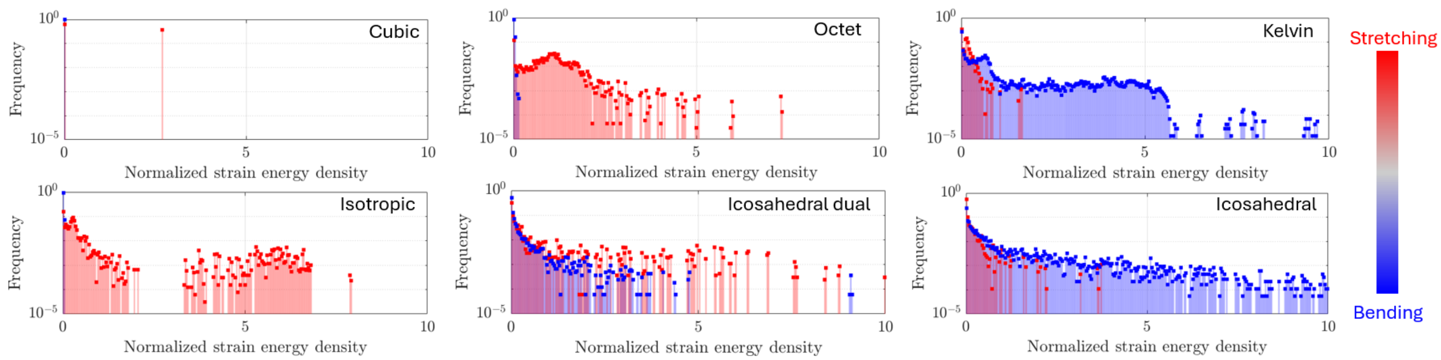

Fig. S3: Distribution of strain energy density separated into bending and stretching contributions for different 3D trusses, complementing the linear-regime results of Figure 3.

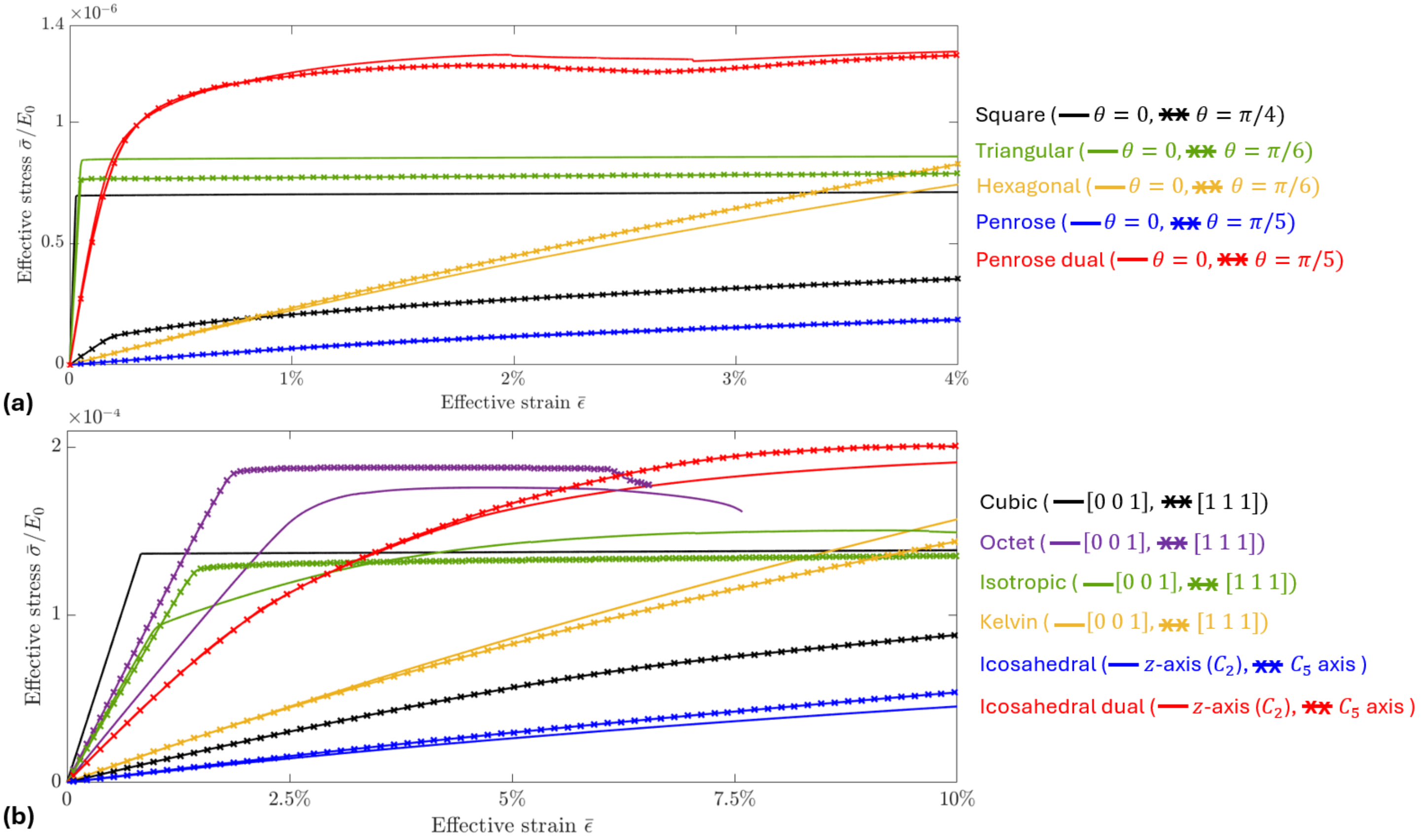

Fig. S4: Nonlinear responses of (a) 2D and (b) 3D trusses with $\rho = 5\%$ for different orientations.

quasicrystals. The cubic lattice presents the most anisotropic response, with the [111] orientation being characterized by bending-dominated behavior (similar to the square lattice). The octet lattice also shows a large anisotropy with distinct linear stiffness and buckling plateaus, with both orientations evidencing a second post-buckling instability due to localized layer collapse. The isotropic lattice is indeed characterized by isotropy in linear stiffness, but evidently different nonlinear responses: compression along the [111] orientation leads to a sharper transition into a buckling plateau at a different stress level in comparison to the smoother transition of the [001] orientation. The Kelvin lattice shows a behavior similar to the hexagonal lattice with deviations in the nonlinear response that steadily increase with strain. Both quasicrystals show a large degree of isotropy, with small deviations for larger strains but approximately direction-independent responses until at least 5% strain.

Supporting note 3: Inelastic Simulations

Material model

Inelastic simulations were conducted within the open-source finite element library *ae108* [8]. These simulations account for material nonlinearity and contact, embedded in a corotational beam framework that accurately captures finite rotations and small strains. Each beam's cross-section is discretized by a number of fibers, which act as integration points and are endowed with a 1D constitutive model and associated internal variables storing information of the history-dependent behavior. The inelastic material model is implemented following the approach of incremental constitutive updates [9]. It accounts for viscoplasticity and viscoelasticity as well as damage. In a nutshell, the chosen damage law reduces the elastic moduli dependent on the most severe status experienced by a fiber's plastic strain. The viscoplastic model of [10] is modified here to also account for viscoelasticity through a generalized Maxwell element with elastic moduli E_∞ and E_j and relaxation times τ_j. The effective incremental potential describing the inelastic material model within each fiber at time step $k+1$ with a time step size Δt, within the framework of

variational constitutive updates, is defined as

$$\begin{aligned}
\mathcal{F}_{\boldsymbol{\varepsilon}^{\mathrm{p},k}}\left(\varepsilon^{k+1},\boldsymbol{\varepsilon}^{\mathrm{p},k+1}\right) =& \overbrace{\frac{1}{2}\left(1-d\right)E_\infty\left(\varepsilon^{k+1}-\varepsilon^{\mathrm{p},k+1}\right)^2}^{\text{elastic energy density}} + \overbrace{\frac{C}{\gamma+1}\left|\varepsilon^{\mathrm{p},k+1}\right|^{\gamma+1}}^{\text{plastic stored energy density}} \\
&+ \overbrace{\sum_{j=1}^{n}\frac{E_j\left(1-d\right)}{2\left(1+\Delta t/\tau_j\right)}\left(\varepsilon^{k+1}-\varepsilon_j^{\mathrm{p},k+1}\right)^2}^{\text{viscoelastic energy density}} \\
&+ \overbrace{\sigma_0\left|\varepsilon^{\mathrm{p},k+1}-\varepsilon^{\mathrm{p},k}\right|}^{\text{dual plastic dissipation potential}} + \overbrace{\frac{\tau_0}{m+1}\frac{\left(\left|\varepsilon^{\mathrm{p},k+1}-\varepsilon^{\mathrm{p},k}\right|\right)^{m+1}}{\left(\Delta t\dot{\varepsilon}_0\right)^m}}^{\text{dual viscoplastic dissipation potential}} \\
&+ \overbrace{\sum_{j=1}^{n}\frac{E_j\tau_j\left(1-d\right)}{2\Delta t\left(1+\Delta t/\tau_j\right)}\left(\varepsilon_j^{\mathrm{p},k+1}-\varepsilon_j^{\mathrm{p},k}\right)^2}^{\text{dual viscoelastic dissipation potential}},
\end{aligned}$$

where – omitting the superscript indicating the timestep – ε represents the total strain, $\boldsymbol{\varepsilon}^{\mathrm{p}} = (\varepsilon^{\mathrm{p}}, \varepsilon_1^{\mathrm{p}}, ..., \varepsilon_n^{\mathrm{p}})$ the vector of internal variables collecting the plastic strain ε^{p} and the internal variables $\varepsilon_j^{\mathrm{p}}$ responsible for the strain accumulated by the jth viscoelastic element. The damage variable d evolves according to the current plastic strain $\varepsilon^{\mathrm{p},k+1}$ and its maximum magnitude experienced during the load history, $\varepsilon_{\max}^{\mathrm{p}}$, according to

$$d = 1 - \exp\left(-s\max\left\{\left|\varepsilon^{\mathrm{p},k+1}\right|, \varepsilon_{\max}^{\mathrm{p}}\right\}\right).$$

For a detailed explanation of the plasticity model and its incremental formulation we refer to [10]. For this study, the model has been calibrated for the UMA 90 polymeric resin through experiments on 3D-printed dogbone samples (thickness 1.6 mm, overall width 9.5 mm, gauge width 6.5 mm, gauge length 25 mm, and overall length 82.5 mm). First, relaxation tests (Figure S5b) allowed for extraction of the viscoelastic model parameters. The viscoelastic behavior is captured well by a Prony series with four branches (Figure S5a). Second, tensile tests up to failure at different strain rates (Figure S5c) were used to calibrate the viscoplastic model parameters. All thus-calibrated parameters of the material model for UMA 90 are reported in Table 1. Contact between beams is modeled with the penalty-based conforming approach, as described in [11], using a penalty coefficient $\epsilon_{\mathrm{N}} = 0.1\ \mathrm{Nm}^{-2}$. The force exchange between elements is accommodated through a finite region of the beams' smoothed center lines, discretized through a set of $n_{\mathrm{QP}} = 4$ quadrature points per segment of contact element.

E_∞	C	γ	σ_0	τ_0	$\dot{\varepsilon_0}$	m	s	ε_f	E_1	E_2	E_3	τ_1	τ_2	τ_3
166 MPa	500 MPa	1.25	0 MPa	5 MPa	0.007 s^{-1}	0.25	16	0.2	447 MPa	259 MPa	216 MPa	6.96 s	110 s	1255 s

Table 1: Calibrated parameters for the UMA 90 material model.

Simulation results

Figure S6(a) presents the numerically obtained stress-strain responses for various topologies, which are directly compared to experimental results in Figure 4 of the main text. The deformed configurations of each design at two significant stages of effective compressive strain are shown in Figures S6(d-g), also highlighting the distribution of plastic strains across each structure. To clearly represent the plastic deformation distribution, these figures include color-coded visualizations of the spatial plastic strain distribution along with histograms depicting the frequency density of plastic strain magnitudes, calculated by averaging over all fibers in the beam cross-sections. From both visual inspection and quantitative analyses, it is evident that each structure exhibits a distinctly different pattern in distributing plastic deformation. The

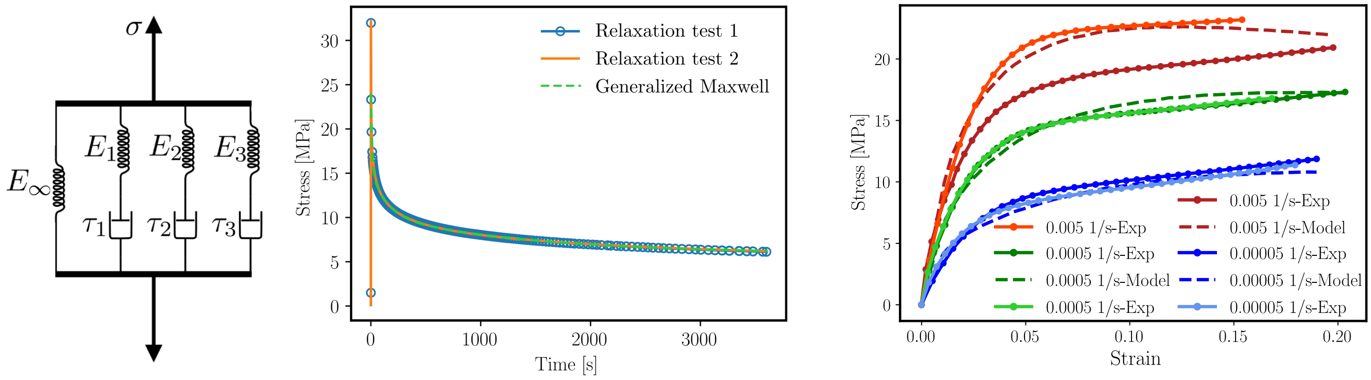

(a) Viscoelastic model (b) Tensile relaxation tests (c) Tensile tests at different strain rates

Fig. S5: UMA 90 material model calibration through experiments on 3D-printed UMA 90 dogbone samples: (a) Generalized Maxwell model, (b) tensile relaxation tests for viscoelastic model calibration with the superimposed fitted model response, (c) tensile tests at different strain rates for viscoplastic model calibration. The two relaxation tests in (b) were conducted by imposing a displacement corresponding to a strain of 2% and measuring the evolution of the tensile force over 1 hour.

periodic, stretching-dominated structures shown in Figures S6(b-d) reveal localized peaks in the plastic strain distribution even at low compressive strains, suggesting a relatively uniform load distribution and plastic deformation—–a behavior that is consistent with periodicity. This pattern aligns with the uniform force chain distributions highlighted in the main text, and it correlates with the observed global buckling and layer collapse mechanisms. In contrast, the stretching-dominated *dual IQC* lattice displays a broader, more distributed plastic strain profile without prominent localized peaks. This distribution indicates that the *dual IQC* structure is protected against global buckling and layer collapse, exhibiting a behavior similar to the bending-dominated *IQC* and *Kelvin* lattices.

The *cubic* lattice specifically demonstrates a clear global buckling mode initiating at approximately 2.5% compressive strain, followed by layer collapse at around 9% effective strain. Unlike in elastic simulations, the introduction of plasticity and associated damage significantly amplifies post-peak softening in the inelastic regime. This softening primarily arises from plastic deformation accumulated uniformly along the buckling columns, with localized concentrations near beam junctions and within collapsing layers. In elastic simulations, beams and junctions typically retain stiffness throughout post-buckling deformation, underscoring a critical difference between elastic and inelastic behavior. Similar mechanisms of global buckling and layer collapse are observed in the *octet* and *isotropic* lattices (Figures S6(c,d)), though the sequence and occurrence of these phenomena vary slightly across topologies and between simulations and experiments. For example, the *cubic* lattice shows consistent global buckling and subsequent layer collapses in both inelastic simulations and experiments, whereas elastic simulations showed only global buckling. Conversely, the *octet* lattice shows global buckling followed by layer collapse in elastic simulations, while experiments and inelastic simulations exhibit layer collapse before global buckling is triggered. The *isotropic* lattice displays global buckling followed by layer collapse in experiments, yet only global buckling without subsequent layer collapse in both elastic and inelastic simulations. These discrepancies are primarily attributed to structural imperfections that affect the overall buckling mode shapes and locations of layer collapses, accounting for some of the variations between simulated and experimental results reported in the main text (Figures 3 and 4).

Despite these differences, it is clear that all periodic stretching-dominated lattices consistently undergo global buckling and/or layer collapses, resulting in unstable deformation patterns that are enhanced by plastic strain and damage accumulation. By contrast, the *IQC dual* lattice (Figure S6(e)) uniquely maintains a distributed and progressive pattern of localized buckling and plastic strain throughout all investigated deformation stages. This absence of global buckling or layer collapse is clearly reflected in its strain distribution plots, which do not exhibit distinct isolated peaks, thereby preserving a significantly superior load-bearing capability. These results collectively highlight the intricate interplay between

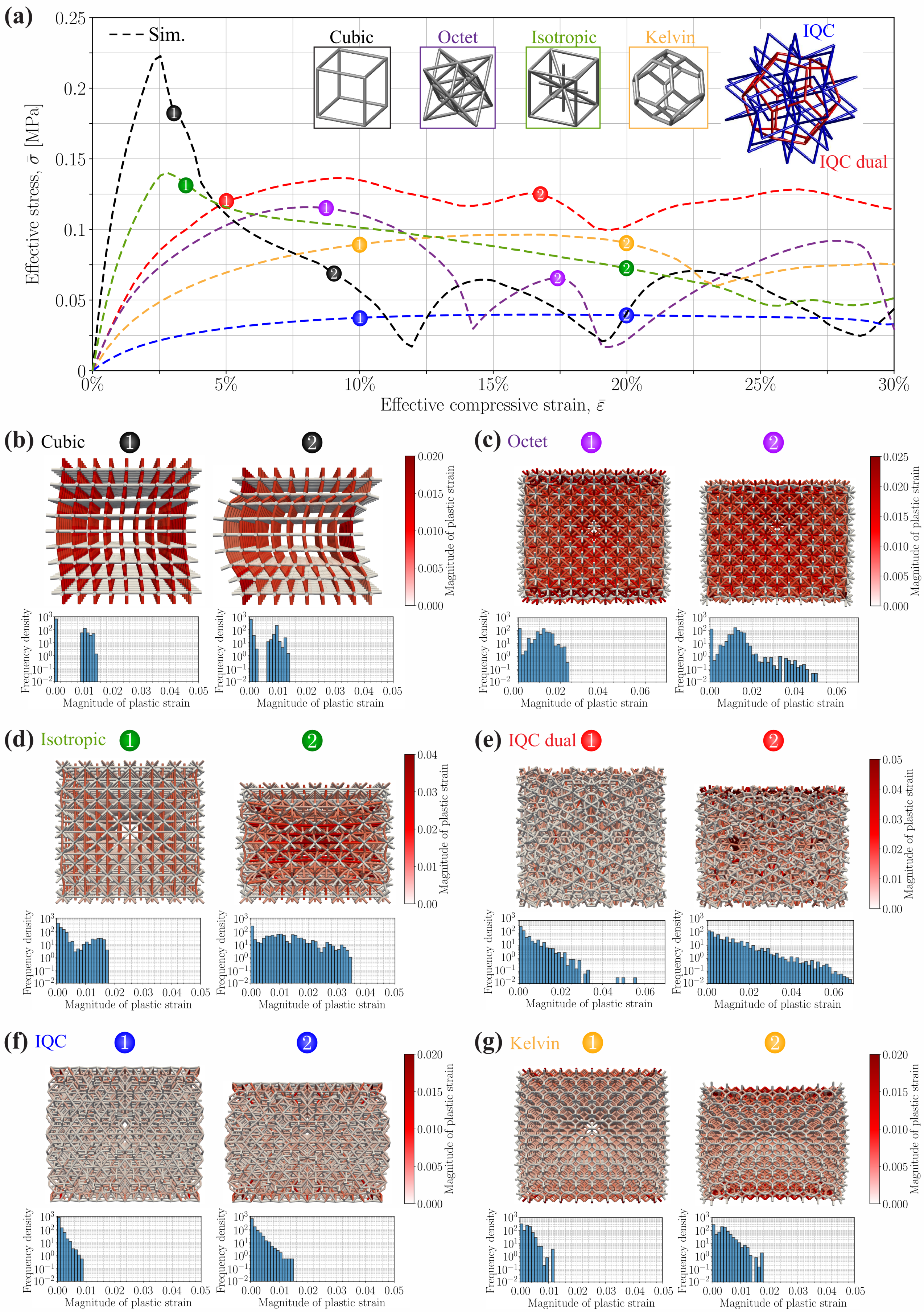

Fig. S6: (a) Simulated stress-strain responses and the corresponding deformation with plastic strain magnitude distribution at the indicated strain levels for the (b) cubic, (c) octet, (d) isotropic, (e) IQC dual, (f) IQC, and (g) Kelvin lattice.

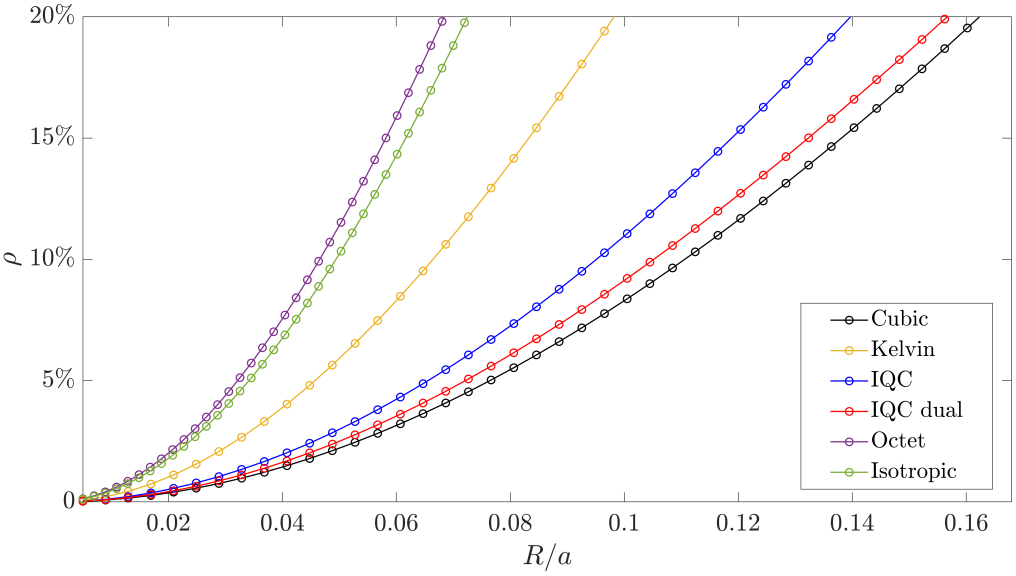

Fig. S7: Variation of relative density ρ with normalized beam radius R/a for different lattice topologies obtained through 3D CAD models.

structural topology, deformation mechanisms, and plasticity. They underline the susceptibility of periodic stretching-dominated lattices to global instability phenomena, while emphasizing the advantageous deformation behavior of the *IQC dual* lattice, which uniquely combines high load-bearing capacity with superior damage tolerance and failure resistance.

Supporting note 4: Geometry of 3D samples

The relationship between relative density and beam radius for different topologies is determined from 3D CAD models, with results illustrated in Figure S7. This relation is used to determine the required beam radius for different relative densities, both in the simulation of 3D trusses, and for the fabrication of samples. In particular, the geometrical parameters employed in the fabricated samples are given in Table 2. Samples were initially tested with $a = 6$mm, such that the cubic domain size of 57mm corresponds to $9.5a$, as in the simulations shown in Figure 3. Since this choice results in different numbers of beams and different beam radii for different topologies, we produced additional samples at an increased unit cell size $a = 8.8$mm for selected periodic lattices, leading to 6.5 unit cells within the considered domain, and an increased beam radius. In contrast, quasicrystalline samples at a reduced length scale of $a = 3.9$mm, leading to a domain size of $14.5a$, were produced with a decreased beam radius. These choices were made to produce re-scaled samples with radii close to 0.4mm, as shown in Table 2. We remark that the radius values reported in Figure S7 and Table 2 for the isotropic sample correspond to the non-diagonal struts, with the diagonal ones being determined by multiplying these values by a factor of 1.14 [12].

Supporting note 5: Experimental results

Figure S8 illustrates the complete experimental stress-strain responses until densification of all specimens tested in this study. The main text summarizes the features of this figure, such as the consistent unstable post-buckling response of stretching-dominated periodic lattices (cubic, octet, isotropic), and the higher

Topology	Cubic	Kelvin	Isotropic	Octet	IQC	IQC dual	Isotropic	Octet	IQC	IQC dual
a [mm]	6	6	6	6	6	6	8.8	8.8	3.9	3.9
R [mm]	0.66	0.4	0.3	0.28	0.57	0.63	0.43	0.41	0.37	0.41

Table 2: Beam radius R and a-values for fabricated specimens.

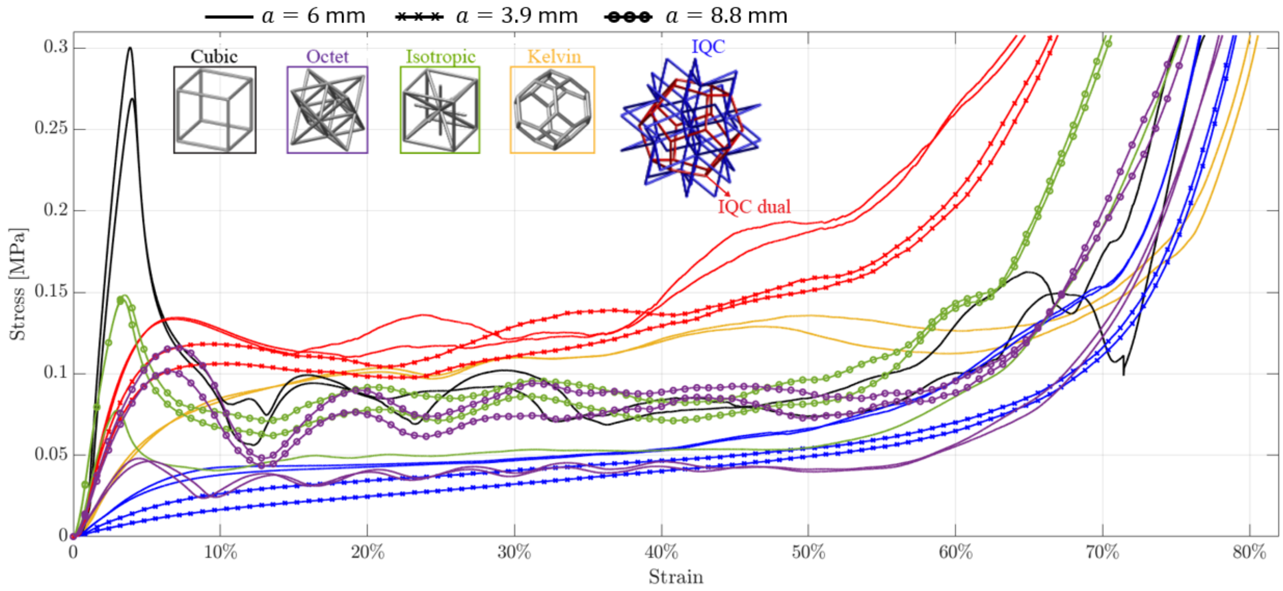

Fig. S8: Experimental stress-strain responses until densification for all samples. Different colors correspond to different topologies, while different line types correspond to the different a-values indicated.

stress plateaus of the icosahedral dual lattices (for both a-values). The full curves are shown in Figure S8. Cyclic stress-strain responses for the Octet and IQC samples are shown in Figure S9 (their property degradation curves are included in Figure 5 of the main text).

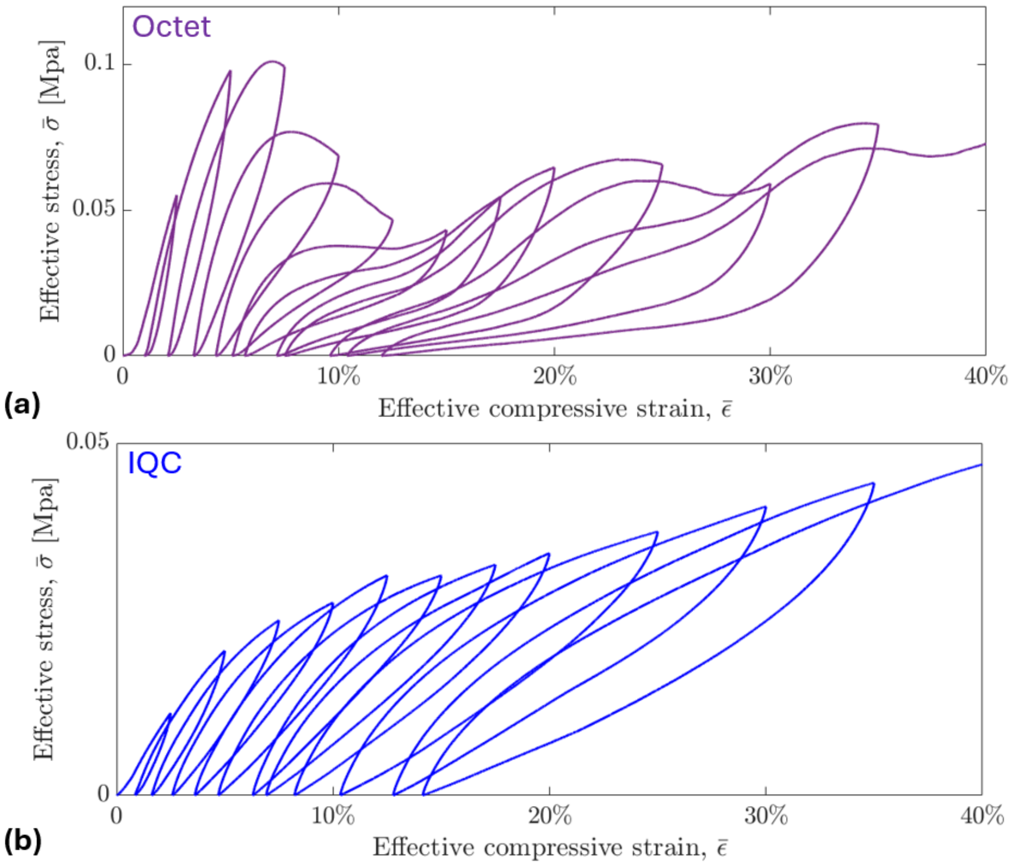

Fig. S9: Experimental cyclic stress-strain responses for Octet (a) and IQC (b) lattices.

References

[1] Joop Los, Ted Janssen, and Franz Gähler. Scaling properties of vibrational spectra and eigenstates for tiling models of icosahedral quasicrystals. *Journal de Physique I*, 3(1):107–134, 1993.

[2] Yi Chen, Muamer Kadic, Sébastien Guenneau, and Martin Wegener. Isotropic chiral acoustic phonons in 3d quasicrystalline metamaterials. *Physical Review Letters*, 124(23):235502, 2020.

[3] Connor T Hann, Joshua ES Socolar, and Paul J Steinhardt. Local growth of icosahedral quasicrystalline tilings. *Physical Review B*, 94(1):014113, 2016.

[4] Joshua D Nolan and Clifford A Reiter. Computing and visualizing three-dimensional quasicrystals. *Fractals*, 26(06):1850093, 2018.

[5] Alexey E Madison. Substitution rules for icosahedral quasicrystals. *RSC Advances*, 5(8):5745–5753, 2015.

[6] Dov Levine and Paul J Steinhardt. Quasicrystals. i. definition and structure. *Physical Review B*, 34(2):596, 1986.

[7] Joshua ES Socolar and Paul J Steinhardt. Quasicrystals. ii. unit-cell configurations. *Physical Review B*, 34(2):617, 1986.

[8] Mechanics and Materials Lab. ae108, 2025. https://doi.org/10.5905/ethz-1007-257 doi:10.5905/ethz-1007-257.

[9] Michael Ortiz and Laurent Stainier. The variational formulation of viscoplastic constitutive updates. *Computer Methods in Applied Mechanics and Engineering*, 171(3):419–444, 1999.

[10] Konstantinos Karapiperis, Kaoutar Radi, Zifan Wang, and Dennis M. Kochmann. A variational beam model for failure of cellular and truss-based architected materials. *Advanced Engineering Materials*, page 2300947, 2023.

[11] Konstantinos Karapiperis, Adrian Widmer, Elias Pescialli, and Dennis M. Kochmann. A conforming frictional beam contact model. *Computer Methods in Applied Mechanics and Engineering*, 431:117310, 2024.

[12] Gérald Gurtner and Marc Durand. Stiffest elastic networks. *Proceedings of the Royal Society A: Mathematical, Physical and Engineering Sciences*, 470(2164):20130611, 2014.
